# The interplay of nitrogen sources and viral communities in the biodegradation of atrazine in agricultural soils

**DOI:** 10.3389/fmicb.2025.1645559

**Published:** 2025-07-16

**Authors:** Yongfeng Wang, Mark Radosevich, Lu Yang, Ying Zhang, Ninghui Xie, Xiaolong Liang

**Affiliations:** ^1^Key Laboratory of Pollution Ecology and Environmental Engineering, Institute of Applied Ecology, Chinese Academy of Sciences, Shenyang, China; ^2^CAS Key Laboratory of Forest Ecology and Silviculture, Institute of Applied Ecology, Chinese Academy of Sciences, Shenyang, China; ^3^Department of Biosystems Engineering and Soil Science, The University of Tennessee, Knoxville, TN, United States; ^4^The Benioff Center for Microbiome Medicine, University of California San Francisco, San Francisco, CA, United States

**Keywords:** atrazine degradation, viral lifestyles, nitrogen amendment, AMGs, top-down control

## Abstract

Atrazine is a widely used herbicide, and its degradation is primarily mediated by microbial activity. However, the interplay between nutrient availability and viral infections on microbial degradation of atrazine remains unexplored. Here, we investigated atrazine degradation under different nitrogen amendments (ammonium, nitrate, and urine) and the influence of soil viruses (intracellular and extracellular viruses). The results showed that atrazine degradation was greater with the addition of extracellular viruses without exogenous nitrogen sources. The added nitrogen sources (nitrate and urine) completely inhibited atrazine degradation. Ammonium impeded atrazine degradation, which was promoted with the addition of intracellular viruses. The metagenomic-based evidence revealed that nitrogen amendments significantly alter bacterial and viral community composition. *Peduoviridae* emerged as the predominant viral family, with its prevalence and temperate phage ratio strongly influenced by nitrogen availability, underscoring the role of nutrient dynamics in shaping virus-host interactions. The presence of viruses selectively enriched atrazine degradation genes and auxiliary metabolic genes (AMGs) associated with key microbial metabolic pathways, revealing potential mechanisms by which viral infections contribute to pollutant biodegradation. The findings highlight the complex interplay between viral predation, microbial adaptation, and nitrogen-driven shifts in microbial community structure and function, offering new perspectives on how viruses shape bioremediation processes in agroecosystems.

## 1 Introduction

Atrazine is one of the most extensively used herbicides in agricultural ecosystems, where its persistence in soil is influenced by prior exposure history, environmental conditions, and intrinsic chemical stability (Jablonowski et al., 2009). As a recognized endocrine disrupter, atrazine poses ecological risks to aquatic vertebrates (Solomon et al., 2008) and potential health hazards to humans, affecting endocrine, central nervous, and immune system functions (Chang et al., 2022). Atrazine dissipates in soil via biotic degradation, primarily mediated by microbial communities (Mili et al., 2022) or abiotic processes such as photodegradation and chemical hydrolysis (Singh et al., 2018). Environmental factors, including temperature, moisture, and nutrient availability, significantly influence the efficiency of these degradation pathways (Chowdhury et al., 2021). Given the ecological and human health concerns associated with atrazine contamination, microbial-based bioremediation techniques have been developed to mitigate its persistence in soil (Chen et al., 2019). The structure and functional capacity of soil microbial communities are critical determinants of atrazine degradation efficiency (Gao et al., 2024). This highlights the need to understand the natural drivers that regulate microbial activity in contaminated environments.

Nitrogen fertilizers and herbicides are frequently co-applied in agroecosystems to enhance crop yields. Nitrogen availability is a key factor in shaping microbial community composition and metabolic activity. Some studies have reported that the presence of a preferred nitrogen source repressed the atrazine degradation (Rhine et al., 2003; Sharma et al., 2019). Microorganisms preferentially assimilate the more accessible nitrogen source when presented with multiple forms of nitrogen. The three primary nitrogen fertilizers (i.e., ammonium, nitrate, and urea) have been found to suppress atrazine degradation to varying degrees for different degrading bacteria (Gebendinger and Radosevich, 1999; García-González et al., 2003). These nitrogen sources can serve as readily available nitrogen sources for microbial growth and metabolism, potentially altering microbial degradation pathways of atrazine (Govantes et al., 2010). However, the extent to which different nitrogen sources modulate microbial-mediated atrazine degradation and community dynamics remains largely unexplored.

Viruses, the most abundant biological entities on Earth, play fundamental roles in shaping the ecology of microbial communities through lytic and lysogenic cycles (Kuzyakov and Mason-Jones, 2018). Viral infections can significantly alter microbial metabolism, community turnover, and nutrient cycling, positioning viruses as key regulators of biogeochemical processes (Liang et al., 2024a). A growing number of studies have indicated the importance of viruses in altering the composition of soil microbial communities. Lytic viruses disrupt microbial populations through host lysis, releasing intracellular nutrients that stimulate the growth of non-infected microbes, a process known as the “viral shunt” (Wommack and Colwell, 2000). In contrast, temperate viruses integrate into host genomes and may enhance microbial fitness under environmental stress through the acquisition and transfer of auxiliary metabolic genes (AMGs) (Jansson and Wu, 2023). AMGs have been implicated in modulating pollutant degradation, either enhancing or inhibiting microbial degradation pathways (Ghosh et al., 2008; Zheng et al., 2022; Chao et al., 2023). Given their dual impact on microbial function, viral infections may either promote or suppress atrazine degradation depending on virus-host interactions and environmental conditions. Despite growing recognition of viral influence in soil microbial ecology, the combined effects of viral infection and nutrient availability on microbial community structure and function remain poorly understood.

In this study, we selected atrazine as a model compound to investigate the interplay between viruses, nitrogen sources, and microbial-mediated herbicide degradation in soil. Specifically, we examined how viral reproductive strategies (lytic vs. temperate) influence atrazine degradation, soil microbial community dynamics, and metabolic functions in the presence of different nitrogen sources (ammonium, nitrate, and urea). By elucidating the role of virus-host interactions in shaping microbial responses to atrazine exposure, this study provides novel insights into viral contributions to soil bioremediation and offers a new perspective on managing herbicide contamination in agricultural ecosystems.

## 2 Materials and methods

### 2.1 Soil sampling

The soil samples were collected from agricultural fields in Liaohe Plain of Northeast China (121°01′E, 42°03′N). The soil type is brown soil. The climate in this area is a temperate semi-humid continental monsoon climate. Surface soil samples (0–20 cm depth) were collected in July 2023 from a maize field with over 10-year history of atrazine application. The soil samples were immediately transported on ice to the lab. Upon arrival, the soil samples were thoroughly mixed by passing through 2 mm sieves to remove straw, roots, and stone.

### 2.2 Bacterial and viral extraction

For the extraction of bacteria and viruses, 10 g of soil was added to 1 L of minimal salts medium (MSM, 1.265 g L^−1^ K_2_HPO_4_, 1.125 g L^−1^ Na_2_HPO_4_, 0.049 g L^−1^ MgSO_4_, and 2.702 g L^−1^ C_6_H_12_O_6_). The mixture was incubated for 24 h at room temperature. The pre-incubation was intended to decrease the preference for exogenous nitrogen sources using the nitrogen-free medium. After incubation, viruses and bacteria were extracted as described previously (Williamson et al., 2003). Briefly, 100 mL of the soil suspension was centrifuged at 9,000 g for 20 min. The supernatant was filtered through a 0.22 μm filter membrane, and the filtrate was regarded as a suspension of extracellular viruses (~100 mL, 2.41 × 10^9^ viral-like particles mL^−1^ based on the epifluorescence direct counting) that were predominantly lytic viruses, although the mixture likely also included some temperate viruses in the lytic mode of replication. The bacterial cells on the filter were washed three times and then resuspended in 100 mL of MSM. For obtaining temperate virus, 20 mL of the bacterial suspension was used for induction assay with the addition of mitomycin C (final concentration of 0.5 μg mL^−1^), and the mixture was incubated at room temperature for 18 h. After incubation, the cell suspension was centrifuged at 9,000 g for 20 min, and the supernatant was filtered through a 0.22 μm filter membrane. The filtrate (~20 mL, 8.28 × 10^8^ viral-like particles mL^−1^ based on the epifluorescence direct counting) was regarded as a suspension of intracellular viruses that primarily consist of temperate viruses that were induced to the lytic mode of replication, recognizing that only those lysogens induced by mitomycin C exposure would be present in the resulting filtrate.

### 2.3 Incubation experiment

In this experiment, MSM with 30 mg L^−1^ atrazine was used to support microbial growth. Various nitrogen sources (50 mg L^−1^ N), including NaNO_3_ (N), (NH_4_)_2_SO_4_ (A), and urea (U), were added to MSM, separately, while no exogenous nitrogen source was added into the medium of the nitrogen-free control treatment (C). The extracted bacterial populations were inoculated into each medium (v/v, 1:58). To investigate how the presence of viruses influenced atrazine degradation, extracellular lytic viruses or induced temperate viruses were added into the medium. A portion of the bacterial suspension and viral suspension was inactivated (autoclaved at 121°C for 20 min, 3 cycles) and added to sterile MSM with atrazine to assess the abiotic transformation of atrazine. Specifically, 58 mL of MSM containing 30 mg L^−1^ atrazine was used for each treatment and loaded in a 150 mL Erlenmeyer flask. Nitrogen sources were added into the medium separately, followed by addition of 1 mL bacterial suspension and 1 mL viral suspension, respectively. The extracellular virus suspension (V_EX_) and intracellular virus suspension (V_IN_) were individually mixed with active bacterial suspension. As a negative control, the same amount of inactivated virus was added into the medium of the virus-free control treatment (V_C_). The incubation experiment was performed on a shaker at 150 rpm at 25°C for 24 days. Each treatment was conducted with three replicates. The bacterial growth, community structure, function, and atrazine biodegradation were monitored throughout the incubation. Samples from each culture were taken for atrazine detection and optical density measurements (at a wavelength of 600 nm; OD600) at 0, 3, 7, 11, 15, and 24 days, respectively. At the end of the incubation, the microbial samples were taken for high-throughput sequencing. The growth of the enrichment cultures was measured with OD600 using a spectrophotometer, and residual atrazine levels were determined using liquid chromatography.

### 2.4 Detection of atrazine

For detection of atrazine in the cultures, the collected culture samples were passed through 0.22-μm Millex syringe filters to remove cells (Merck Millipore Ltd., Tullagreen, Co. Cork, Ireland). Preliminary tests indicated that atrazine did not adsorb to the filters or the filter apparatus. Atrazine in the filtrate was determined by Thermo UltiMate 3000 (Thermo Scientific, United States) equipped with a diode array detector. The injection volume was 20 μL, and atrazine and/or its metabolites were separated on a Kinetex C18 column (Phenomenex Torrance, CA) eluted with a mobile phase consisting of 70% methanol and 30% water (V/V) with a flow rate of 1 mL min^−1^. Detection of atrazine or metabolites was monitored at 240 nm and quantified by the external standard curve that was linear between 0.15 to 30 mg L^−1^. The recovery of atrazine in the sterile controls was near 100%, indicating negligible loss of atrazine by mechanisms other than biodegradation.

The metabolites of atrazine were determined by a UHPLC-UltiMate 3000 system coupled to a Q-Exactive Orbitrap tandem mass spectrometer (Thermo Scientific, United States). The following conditions were used in the selected reaction monitoring (SRM) scan mode: spray voltage at 4,000 V for positive polarity, capillary temperature at 320°C, auxiliary gas heating temperature at 300°C, sheath gas pressure at 30 psi, auxiliary gas pressure at 10 psi. Full scan data collection was executed from m/z 80 to 600. Atrazine and metabolites were separated with Waters Atlantis T3 column (2.1 × 150 mm, 3 μm) at 25°C with a binary mobile phase in isocratic elution mode comprising 70% methanol and 30% water. The flow rate was 0.5 mL min^−1^ and the injection volume was 5 μL.

### 2.5 High-throughput sequencing

#### 2.5.1 16S rRNA amplicon sequencing

The total DNA in each sample was extracted using PowerLyser PowerSoil DNA isolation kit (Qiagen, Hilden, Germany). 16S rRNA gene (V3-V4 region) was amplified using specific primer 338F and 806R. Sequencing libraries were generated using NEBNext® Ultra™ II DNA Library Prep Kit for Illumina® (New England Biolabs, MA, United States). 16S rRNA amplicon sequencing was performed on an Illumina Nova6000 platform by Guangdong Magigene Biotechnology Co. Ltd. (Guangzhou, China). The raw read data was deposited with NCBI SRA (PRJNA1176382). The 16S rRNA gene sequencing data analysis was performed using QIIME2. The raw sequence data files with forward and reverse reads were combined. DADA2 was used for quality control and feature table construction (Callahan et al., 2016). The high-quality amplicon sequences were aligned to SILVA v138 reference database for taxonomy assignment.

#### 2.5.2 Metagenomic sequencing

Soil samples were sent to Guangdong Magigene Biotechnology Co. Ltd. (Guangzhou, China), and DNA was extracted using ALFA-SEQ Advanced Soil DNA Kit (Findrop, Guangzhou, China) according to the manufacturer’s instructions. Sequencing libraries were generated using ALFA-SEQ DNA Library Prep Kit (Findrop, Guangzhou, China) following manufacturer’s recommendations and index codes were added. The library quality was assessed on the Qubit 4.0 Fluorometer (Life Technologies, Grand Island, NY) and Qsep400 High-Throughput Nucleic Acid Protein Analysis System (Houze Biological Technology Co, Hangzhou, China). Genomic DNA was sequenced on an Illumina NovaSeq 6000 platform and generated 150 bp paired-end reads. Raw sequencing data were deposited in the NCBI Sequence Read Archive (SRA) under accession number PRJNA1177983. Raw reads were subjected to quality control using Trimmomatic (v0.40) (Bolger et al., 2014). Assembly of clean sequence data was performed using MEGAHIT (v1.0.6) (Li et al., 2015), and the Scaftigs (≥500 bp) were assembled from the pooled samples. Open reading frame (ORF) prediction was performed using MetaGeneMark (v3.38) (Zhu et al., 2010). CD-HIT (v4.7) was used to remove redundancy and obtain the unique initial gene catalog (the genes here refer to the nucleotide sequences coded by unique and continuous genes), which were clustered based on the identity of 95% and coverage of 90% (Fu et al., 2012). The clean data of each sample was mapped to the initial gene catalog using BBMap (v38.90) (Bushnell, 2015). Taxonomy prediction and functional annotation were performed by aligning query sequences against the NCBI NR (Non-Redundant) protein sequence database and KEGG (Kyoto Encyclopedia of Genes and Genomes) database using DIAMOND (v0.9.25) (Buchfink et al., 2015).

### 2.6 Identification of viral contig and AMGs

Metagenome-assembled contigs larger than 2,000 bp were selected to identify viral communities and potential AMGs. These data were analyzed using a combination of four tools, including DeepVirFinder (v1.1) (Ren et al., 2020), VIRSorter2 (v2.0) (Guo et al., 2021), and VIBRANT (v1.2.1) (Kieft et al., 2020). CheckV (v0.7.0) was used to assess the quality and completeness of the taxonomic identifications (Nayfach et al., 2021). PhaBOX (v202303) was used for virus species annotation, lifestyle identification, and host prediction (Shang et al., 2023). For auxiliary metabolic genes (AMGs), VIBRANT (v1.2.1) was used to identify virus-encoded AMGs.

### 2.7 Statistical analysis

Microbial community was summarized into *α*-diversity, *β*-diversity, taxonomic changes and functional pathway analysis. Shannon diversity indices (measuring within-sample diversity) were calculated based on the rarefied data. ANOVA was applied to test differences between groups, with a *p*-value less than 0.05 indicating statistical significance. β-diversity characterizes the shared diversity between bacterial populations in terms of ecological distance. To visualize the relationships among samples, principal coordinate analysis (PCoA) with Bray-Curtis distance method was applied. Permutational multivariate analysis of variance (PERMANOVA) was used for testing the association with β-diversity (“adonis” function in R package “vegan,” 999 permutations). Core microbiomes and metabolic functions were identified by stringent measures based on presence/absence patterns at the genus level and KEGG L3 and were visualized using the “pheatmap” package. All figures were prepared using ggplot2 package.

## 3 Results

### 3.1 Atrazine degradation as influenced by viral infection and nitrogen amendments

**Figure 1:**
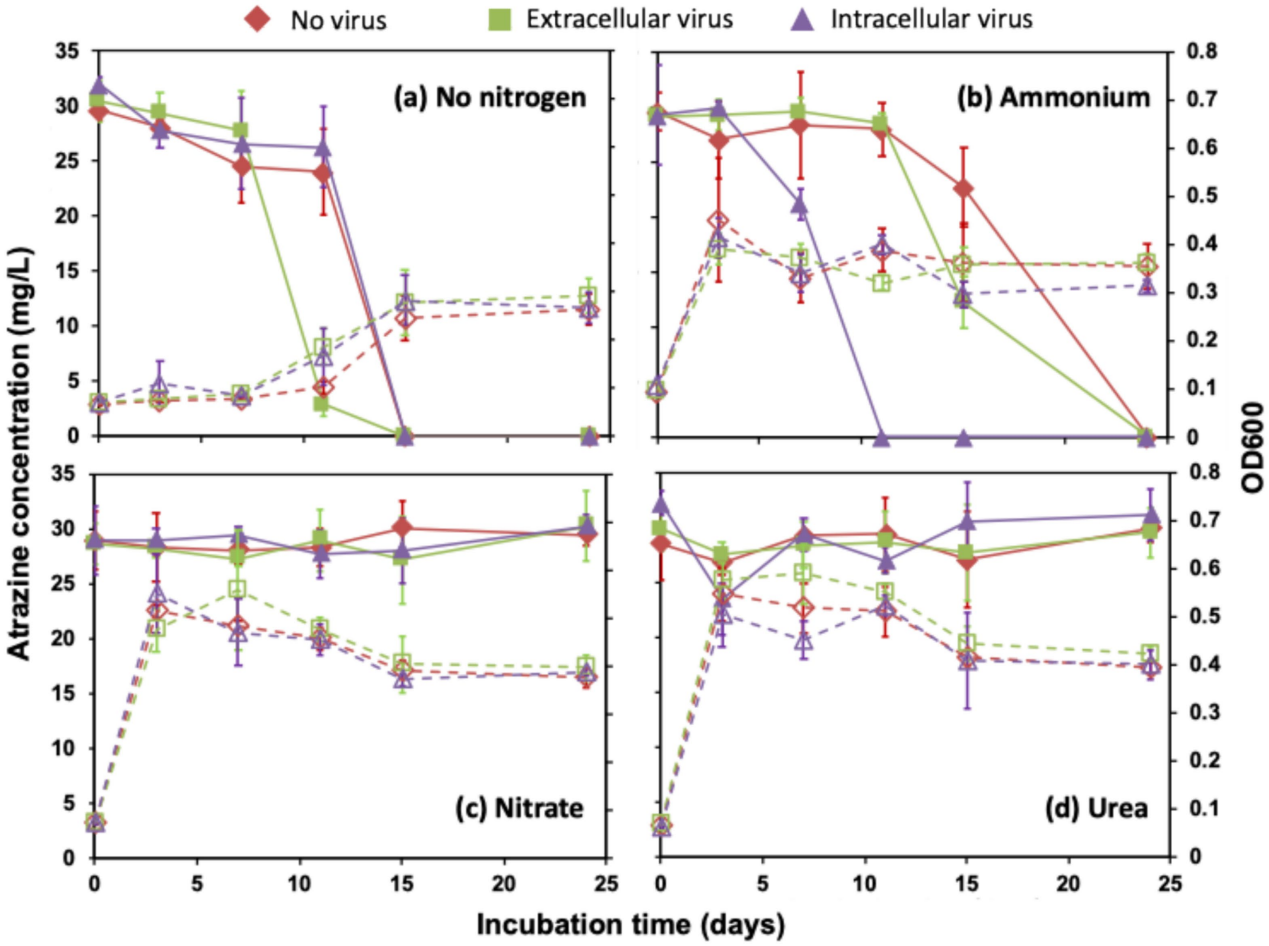
Atrazine degradation (solid line, left axis) and the microbial growth dynamics (dash line, right axis) with different virus amendments (i.e., no virus, extracellular virus, and intracellular virus) under the conditions of different nitrogen sources, including no exogenous nitrogen source **(a)**, ammonium **(b)**, nitrate **(c)**, and urea **(d)**. All data show the mean of three replicates with standard deviation.

In the absence of viral amendments and exogenous nitrogen sources, atrazine degradation exhibited a delayed onset but proceeded rapidly after 11 days, leading to complete degradation within 15 days of incubation (Figure 1a). When ammonium was added as an amendment, atrazine degradation was slightly delayed and completed after 24 days (Figure 1b). In contrast, atrazine degradation was completely inhibited with nitrate and urea amendments over the incubation period (Figures 1c,d).

When extracellular viruses were introduced into the treatment without an exogenous nitrogen source, atrazine degradation was significantly accelerated (Figure 1a). The atrazine concentration dropped from 27.8 mg L^−1^ on the 7th day to 2.9 mg L^−1^ on the 11th day, which was significantly lower than that in the absence of viruses. It was completely removed after 15 days of incubation. The presence of ammonium, in combination with extracellular viruses, extended atrazine degradation to 24 days, similar to the ammonium-only treatment (Figure 1b). Notably, nitrate and urea amendments continued to suppress degradation, reinforcing their inhibitory role irrespective of viral influence (Figures 1c,d).

Under intracellular virus amendments, atrazine degradation patterns closely resembled those of the virus-free and nitrogen-free conditions (Figure 1a). However, with ammonium supplementation, atrazine degradation was significantly accelerated, initiating on the 3rd day and completing within 11 days (Figure 1b). This suggests that intracellular viral activity, possibly through host metabolic reprogramming, enhanced microbial utilization of atrazine in the presence of ammonium. In contrast, nitrate and urea consistently inhibited atrazine degradation under all conditions, underscoring their suppressive effects (Figures 1c,d).

In the sterilized treatment, no atrazine degradation occurred (Supplementary Figure S1), confirming the absence of abiotic degradation. Two transformation products, i.e., hydroxyatrazine and deethylatrazine, were identified in all the treatments that exhibited loss of atrazine, suggesting enzymatically catalyzed dechlorination and dealkylation reactions were functional in the enrichment cultures (Supplementary Figure S2). No additional degradation byproducts were detected, suggesting a conserved metabolic pathway for atrazine breakdown in the studied microbial communities.

### 3.2 Microbial growth response to nitrogen amendments

Microbial growth was mainly affected by nitrogen availability rather than viral amendments (Figure 1). The microbial growth exhibited a long lag phase (~10 days) in the MSM that had no exogenous nitrogen sources. The microbial growth in the MSM without the supply of exogenous nitrogen source yielded low biomass (OD600 ranging from 0.26 to 0.29). The synchronization of microbial growth initiation and atrazine degradation suggests that atrazine can be used as a nitrogen source to sustain microbial growth and proliferation. In the presence of exogenous nitrogen sources, the microbial growth exhibited accelerated kinetics relative to nitrogen-free control and reached the stationary phase earlier than under nitrogen-limited conditions. On the 3rd day of incubation, the biomasses were greatest with the addition of the exogenous nitrogen sources, and the growth was less than in the medium with ammonium (OD600 ranging from 0.39 to 0.45) than that with nitrate (OD600 ranging from 0.48 to 0.55) and urea (OD600 ranging from 0.51 to 0.58). For the sterilized treatment, no microbial growth was observed (Supplementary Figure S1), confirming that all observed biomass increases were biologically driven.

### 3.3 Influence of viruses and nitrogen sources on microbial community structure

**Figure 2:**
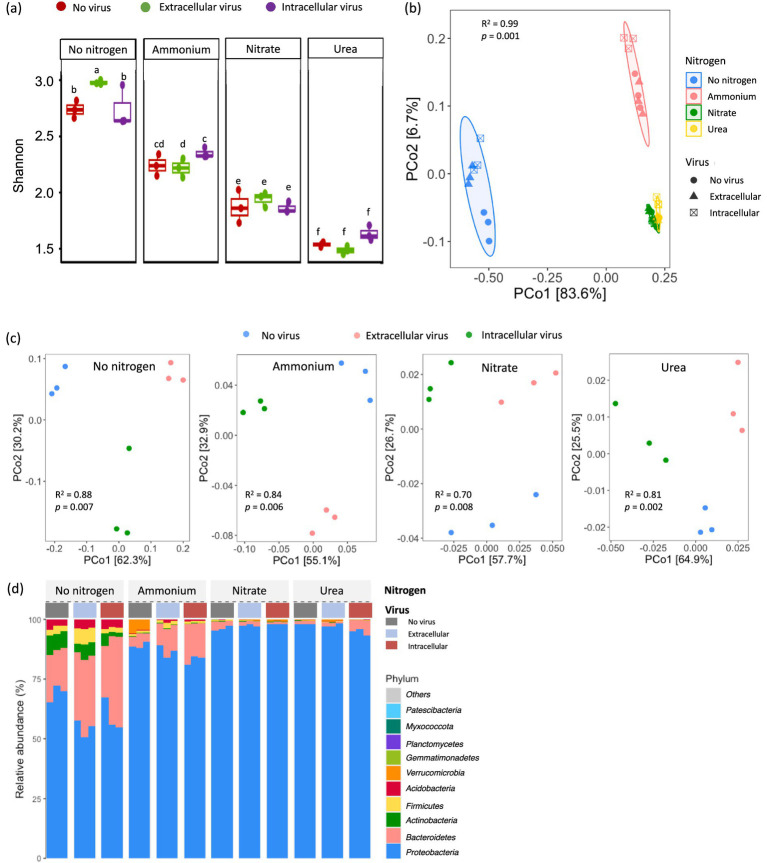
Effects of exogenous nitrogen sources and viral addition on bacterial community diversity and composition. **(a)** Alpha diversity measured by the Shannon index for bacterial communities across different nitrogen conditions (No nitrogen, Ammonium, Nitrate, and Urea) with three viral treatments (No virus, Extracellular virus, and Intracellular virus). Identical alphabetic characters indicate no significant difference between groups, whereas different letters denote statistically significant differences (Tukey’s HSD, *p* < 0.05). **(b)** Principal Coordinates Analysis (PCoA) based on Bray–Curtis dissimilarity illustrating the clustering of bacterial communities primarily by nitrogen source (PERMANOVA, *p* = 0.001). **(c)** PCoA plots showing the impact of viral addition within each nitrogen condition. **(d)** Relative abundance of top 10 microbial phyla with different viruses and nitrogen sources.

To characterize the linkage between the atrazine degradation and microbial community structure, 16S rRNA gene amplicon and metagenomic sequencing were conducted. Based on 16S rRNA gene amplicon sequencing, the alpha diversity of the bacterial communities, as measured by the Shannon index, decreased significantly upon the addition of exogenous nitrogen sources to the MSM cultures (*p* < 0.05; Figure 2a). The observed diversity following a decreasing trend: No nitrogen > Ammonium > Nitrate > Urea. Furthermore, in nitrogen-free conditions, the Shannon index increased with the addition of extracellular viruses (*p* < 0.05; Figure 2a). Additionally, in ammonium-amended treatments, intracellular viruses promoted microbial diversity (*p* < 0.05). These findings suggest that both nitrogen availability and viral infections modulate bacterial diversity in atrazine degradation. The PCoA based on Bray–Curtis dissimilarity revealed that bacterial community composition was primarily driven by nitrogen source availability rather than the viral amendments (PERMANOVA, *p* < 0.01; Figure 2b). The viral amendments with either intracellular or extracellular viruses had a significantly different influence on the bacterial community structure under varying nitrogen conditions (PERMANOVA, *p* < 0.01; Figure 2c). This highlights the secondary yet notable role of viruses in shaping microbial community succession.

At the phylum level, the bacterial communities were dominated by *Proteobacteria* (Figure 2d). The relative abundance of *Proteobacteria* in the treatment of nitrogen-free and ammonium-enriched culture, respectively, ranged from 51 to 72% and from 81 to 91%, while the relative abundance of *Proteobacteria* in the treatment with nitrate and urea ranged from 93 to 98%. Other major bacterial phyla included *Bacteroidetes*, *Actinobacteria*, *Firmicutes*, *Acidobacteria, Verrucomicrobia, Gemmatimonadetes, Planctomycetes, Myxococcota*, and *Patescibacteria*. The difference in the relative abundance of these taxa among the different treatments suggested that nitrogen sources significantly influenced the bacterial community composition based on the conclusion of the incubation experiment. Notably, *Bacteroidetes, Actinobacteria, Firmicutes*, and *Acidobacteria* were significantly enriched in the nitrogen-free control, of which *Bacteroidetes* accounted for more than 15%. Moreover, the relative abundance of *Bacteroidetes* in the treatment with ammonium (3–17%) was more than that in nitrate and urea enrichment cultures. Interestingly, a high relative abundance of *Verrucomicrobia* was detected ranging from 4 to 6% in the treatment with ammonium and without viruses, but it was not detected in the treatment with ammonium and viruses. This result suggested that viral activity, substrate competition, or toxic metabolite production effectively eliminated *Verrucomicrobia*.

Based on 16S rRNA gene amplicon sequencing, no significant change in bacterial composition and no obvious atrazine degradation were observed in nitrate and urea enrichment cultures; thus, metagenomic sequencing was not conducted in these treatments. The metagenomic sequencing data derived from microbial community composition in treatments of nitrogen-free and ammonium enrichment culture (Supplementary Figure S3) were similar to those derived from 16S rRNA gene amplicon sequencing data (Figure 2d). The microbiota consisted mainly of *Proteobacteria* (>81%), *Bacteroidetes* (2.0–9.4%), *Actinobacteria* (1.7–6.0%), *Firmicutes* (0.9–3.4%), and *Acidobacteria* (0.6–1.6%). *Proteobacteria* exhibited the greatest relative abundance across all treatments. The relative abundance of *Verrucomicrobia* was particularly high in the treatment with ammonium and without viruses (>2%), but no *Verrucomicrobia* was detected in the treatment with ammonium and viruses.

**Figure 3:**
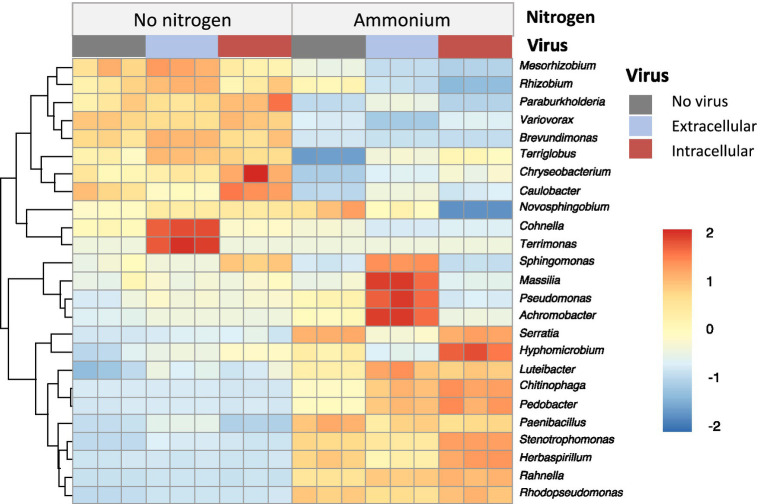
The heatmap for the top 25 bacterial genera without the addition of nitrogen source and with the addition of ammonium based on 16S rRNA gene amplicon sequencing.

To elucidate the effects of viral amendments and nitrogen sources on microbial community structure, the significant differences of the main bacterial genera were assessed after incubation (*p* < 0.05; Figure 3). The most dominant genera in the treatment without exogenous nitrogen amendment included *Terriglobus*, *Caulobacter*, *Mesorhizobium*, *Rhizobium*, *Paraburkholderia*, *Variovorax*, *Brevundimonas*, *Novosphingobium*, *Cohnella*, and *Terrimonas*, while the most dominant genera in the ammonium-enriched culture included *Sphingomonas*, *Massilia*, *Pseudomonas*, *Achromobacter*, *Serratia*, *Hyphomicrobium*, *Paenibacillus*, *Rahnella*, *Rhodopseudomonas*, *Stenotrophomonas*, *Herbaspirillum*, *Luteibacter*, *Chitinophaga*, and *Pedobacter* (Figure 3).

### 3.4 Viral community composition and virus-host interactions

**Figure 4:**
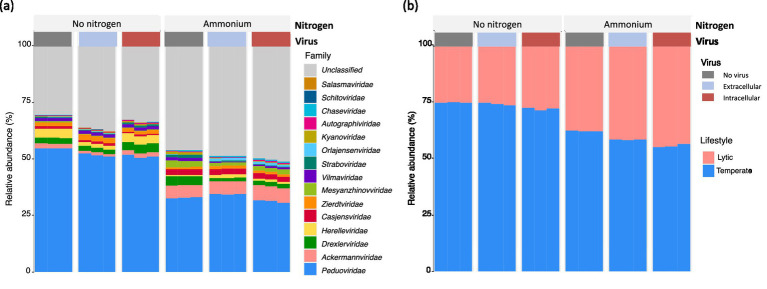
Relative abundance of virus family **(a)** and lifestyle **(b)** without the addition of nitrogen source and with the addition of ammonium based on metagenomic sequencing.

Based on the metagenomic sequencing, *Uroviricota* was the only virus phylum with the relative abundance of less than 0.1% (total sequences) (Supplementary Figure S3). Further, we analyzed the DNA viral community with a total of 2,679 viral contigs from the metagenomic assemblies, where 1,247 viral contigs were ascribed to the phage. Among them, 431 phage contigs were taxonomically annotated to the family level. The viral families included *Peduoviridae, Ackermannviridae, Drexlerviridae, Herelleviridae, Casjensviridae, Zierdtviridae, Mesyanzhinovviridae, Vilmaviridae, Straboviridae, Orlajensenciridae, Kyanoviridae, Autographivirdae, Chaseviridae, Schitoviridae, and Salasmaviridae* (Figure 4a). *Peduoviridae* were the most dominant family within all viromes (Figure 4a). The relative abundance of *Peduoviridae* in the nitrogen-free treatment (50–55%) was much higher than the ammonium-enriched treatment (31–34%). Moreover, the ratio of temperate phages in the nitrogen-free treatment (71–75%) was also much higher than the ammonium-enriched treatment (55–62%) (Figure 4b). The difference in the relative abundance of these taxa among the different treatments suggested that nitrogen sources significantly influenced the viral community composition and lifestyles. Interestingly, despite the observed shifts in bacterial community structure, the addition of extracellular or intracellular viruses had no significant impact on the overall composition or lifestyle of the viral community.

**Figure 5:**
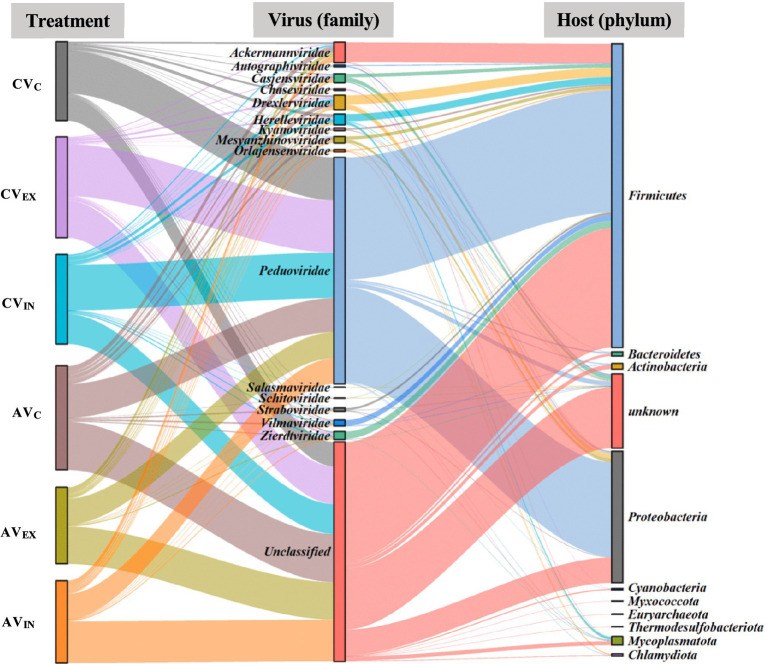
Predicted virus (family level) and host (phylum level) association. CV_C_, CV_EX_, and CV_IN_ denote no virus, extracellular and intracellular virus without exogenous nitrogen other than atrazine; AV_C_, AV_EX_, and AV_IN_ denote no virus, extracellular and intracellular virus with ammonium amendments.

Virus-host interactions were predicted, revealing that viral contigs were associated with 10 bacterial phyla including *Firmicutes, Bacteroidetes, Actinobacteria, Proteobacteria, Cyanobacteria, Myxococcota, Euryarchaeota, Thermodesulfobacteriota, Mycoplasmatota,* and *Chlamydiota* (Figure 5). *Firmicutes* and *Proteobacteria* were the predominant predicted bacterial hosts for *Peduoviridae*, underscoring their potential susceptibility to viral infections in the enrichment cultures.

### 3.5 Viral influence on microbial functional genes and metabolic pathways

**Figure 6:**
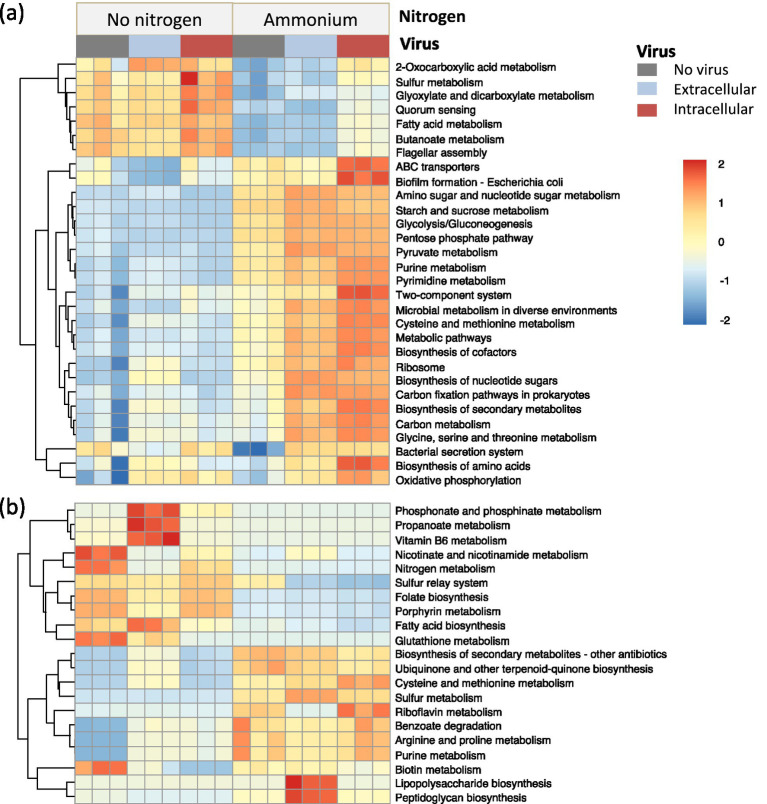
The heatmap for KEGG ortholog (L3) pathways of top 30 microbial functions **(a)** and virus-encoded AMGs **(b)** based on metagenomic sequencing.

**Figure 7:**
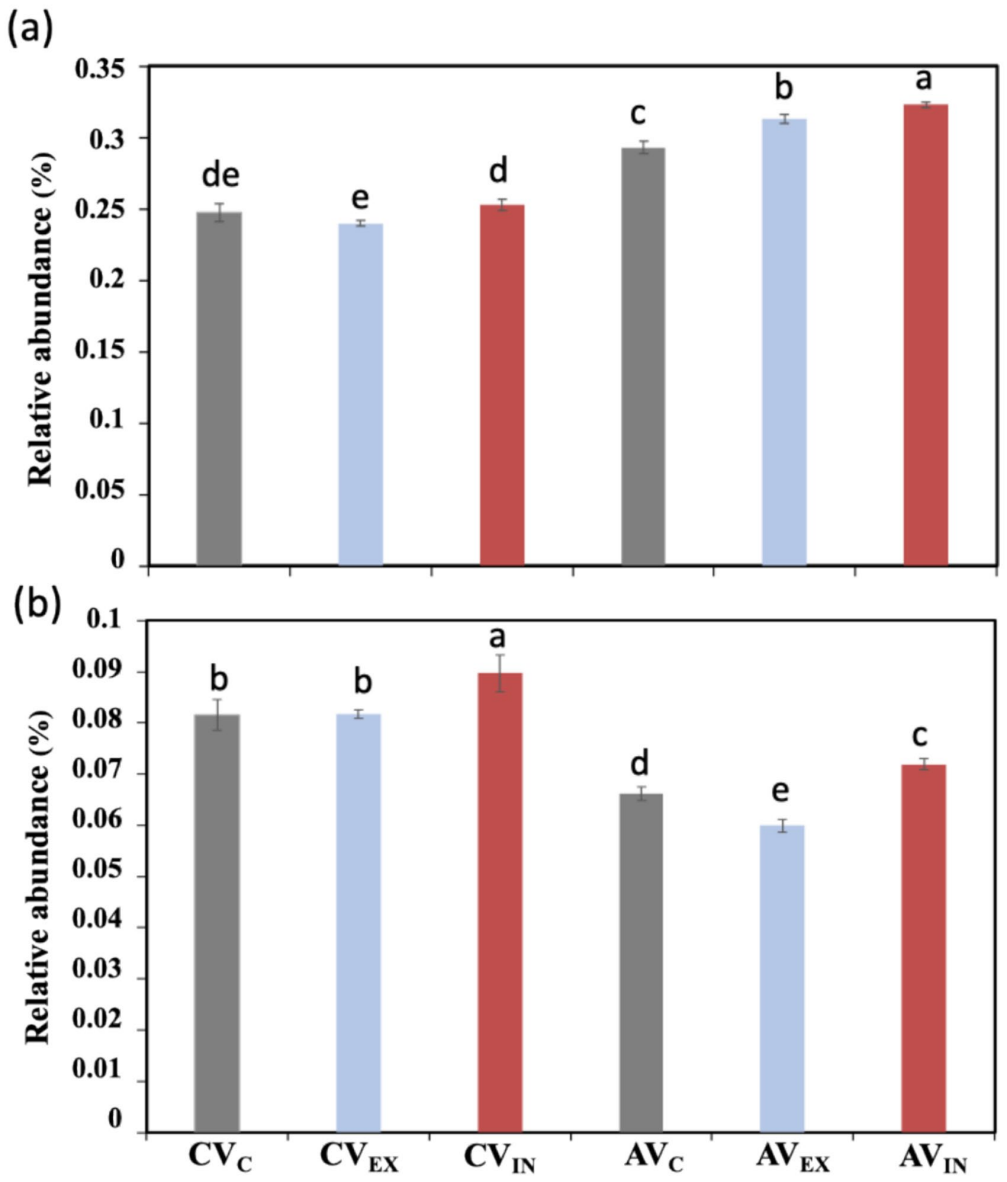
Relative abundance of nitrogen metabolism **(a)** and atrazine degradation genes **(b)**. CV_C_, CV_EX_, and CV_IN_ denote no virus, extracellular and intracellular virus without exogenous nitrogen other than atrazine; AV_C_, AV_EX_, and AV_IN_ denote no virus, extracellular and intracellular virus with ammonium amendments. Data show the mean of three replicates with standard deviation, and different lowercase letters among treatments denote significant differences (ANOVA, *p* < 0.05).

To further study the linkage between atrazine degradation and microbial metabolic potential, the bacterial functional genes and metabolic pathways in the enrichment culture metagenomes were predicted by KEGG annotation. Some common functional pathways were observed across all treatments, while most functional pathways varied based on the presence of viruses and nitrogen sources (*p* < 0.05; Figures 6a, 7a). With the addition of intracellular viruses, the abundance of genes associated with carbon and nitrogen metabolism increased. In the absence of a nitrogen source other than atrazine, with the addition of intracellular viruses, the abundance of genes involved in sulfur metabolism, glyoxylate, and dicarboxylate metabolism, quorum sensing, fatty acid metabolism, and flagellar assembly increased (Figure 6a). These pathways may play essential roles in microbial adaptation to atrazine degradation under nitrogen-starved conditions. In the presence of ammonium plus intracellular viruses, the relative abundance of ABC transporters, biofilm formation, purine metabolism, pyrimidine metabolism, two-component system, cysteine and methionine metabolism, metabolic pathways, biosynthesis of cofactors, biosynthesis of secondary metabolites, carbon metabolism, glycine, serine and threonine metabolism, and biosynthesis of amino acids increased. Additionally, genes directly involved in atrazine degradation were detected in whole-community DNA metagenomes, including *atzB*, *atzC*, *atzD*, *atzE*, and *atzF* (Figure 7b; Supplementary Figure S4). The genes for the initial step of atrazine degradation, i.e., *atzA* and *trzN*, were not detected in any of the treatments. The addition of intracellular viruses increased the relative abundance of genes associated with atrazine degradation (Figure 7b; Supplementary Figure S4).

A total of 81 AMGs were predicted across the viral genomes, associated with 21 distinct metabolic pathways (Figure 6b). Among them, the dominant pathways included cysteine and methionine metabolism, sulfur relay system, porphyrin metabolism, folate biosynthesis, and sulfur metabolism (Supplementary Figure S5). In the absence of a nitrogen source other than atrazine, the addition of intracellular viruses increased the relative abundance of genes involved in cysteine and methionine metabolism (Figure 6b). In the presence of ammonium, the addition of intracellular viruses increased the relative abundance of genes involved in sulfur relay system, porphyrin metabolism, and folate biosynthesis (Figure 6b).

## 4 Discussion

### 4.1 Inhibited atrazine biotransformation in the presence of added nitrogen sources

The atrazine degradation was primarily mediated through microbial biotransformation processes. Previous studies have revealed that atrazine-degrading microorganisms commonly utilize this herbicide as a nitrogen and/or carbon source for growth (Radosevich et al., 1995; Sharma et al., 2019). However, it has been demonstrated in monocultures, enrichment cultures, and whole soils (microcosm and field scale studies) that exogenous nitrogen sources can repress atrazine biodegradation (Gebendinger and Radosevich, 1999; Abdelhafid et al., 2000; Govantes et al., 2009). In line with these findings, our results demonstrate that the presence of ammonium, nitrate, and urea delayed or completely suppressed atrazine degradation. Ammonium, as a preferred bioavailable nitrogen source, was easily utilized prior to atrazine by microorganisms (Sharma et al., 2019). Once ammonium was consumed, atrazine could be effectively utilized as an alternative nitrogen source, showing the resilience and adaptability of the multispecies enrichment cultures (Rhine et al., 2003). Although ammonium and other nitrogen sources can inhibit atrazine degradation (Abdelhafid et al., 2000; Rhine et al., 2003), some atrazine-degrading bacteria, such as *Agrobacterium radiobacter* J14a, *Pseudomonas* sp. strain ADP, and *Klebsiella* sp. A1, maintain atrazine degradation constitutively even in the presence of exogenous nitrogen sources (Struthers et al., 1998; Bichat et al., 1999; Yang et al., 2010). In our study, however, the enrichment cultures showed clear sensitivity to nitrogen amendments, suggesting that the available nitrogen sources repressed the atrazine-degradative pathways. Interestingly, in cultures amended with nitrate and urea, enhanced microbial growth indicated that increased nutrient availability could contribute to the inhibition of atrazine degradation by suppressing the expression of degradation pathways. In contrast, in ammonium-amended cultures, bacterial growth slowed once ammonium and/or glucose were exhausted, potentially allowing for the reactivation of the atrazine-degrading pathways due to nutrient scarcity.

### 4.2 Selectively enhanced atrazine biotransformation in the presence of added viruses

Viral infection can affect the microbial host community composition and metabolic efficiency (Liang et al., 2024b). Here, our findings reveal that viral amendments, both extracellular (lytic) and intracellular (temperate), selectively accelerated atrazine degradation in the enrichment cultures. The differential impact of lytic and temperate viruses may be attributed to their distinct interactions with microbial host communities. Lytic viral activity can alter the host community composition directly through selective host-cell lysis and indirectly from the release of nutrients derived from lysed cells (Kuzyakov and Mason-Jones, 2018; Wang et al., 2022). Moreover, if the atrazine-degrading bacteria were lysed, the atrazine-degrading enzymes may be released to enhance the atrazine degradation. In nitrogen-limiting conditions, the lytic virus may improve the nutrient level to stimulate microbial activity and community turnover by “viral shunt” (Huang et al., 2024). Alternatively, the temperate virus may have enhanced the metabolism of microbial hosts by virus-encoded AMGs. The expression of AMGs may drive the nutrient cycles, such as carbon, sulfur, and phosphorus metabolism, and have also been found to affect the degradation of organic pollutants (Sun et al., 2023). Here, the temperate virus may have facilitated bacterial metabolism (as evidenced by the significantly increased abundance of functional genes; Figure 7) and accelerated ammonium consumption in the enrichment culture. With the exclusion of ammonium from the medium, atrazine was utilized as a nitrogen source by atrazine-degrading bacteria. Thus, the temperate virus may have decreased the adaptation time and enhanced the atrazine degradation. These results highlighted the complex interplay among atrazine degradation, the interaction of virus and host, and nutrient cycling.

### 4.3 The interplay effects of nitrogen sources and viruses on bacterial and viral communities

The composition and diversity of the microbial community varied with the types of nitrogen sources and viruses added to the cultures. Specifically, in treatments with nitrate and urea, the microbial community was dominated by *Proteobacteria* (>93%; Figure 2d), with significantly reduced diversity compared to treatments of nitrogen-free or ammonium-enriched cultures. This shift in community structure likely inhibited the growth of atrazine-degrading bacteria, consequently suppressing atrazine degradation. The observed dominance of *Proteobacteria*, coupled with reduced microbial diversity, aligns with previous studies, which have suggested that such microbial shifts might negatively impact pollutant degradation (Liu et al., 2020; Liu et al., 2023). Notably, our study revealed that some dominant genera in nitrogen-free treatment were identified that have been recognized previously to include atrazine-degrading bacteria such as *Arthrobacter* (Billet et al., 2019; Cao et al., 2021), *Rhizobium* (Liu et al., 2023), *Paraburkholderia* (Zhao et al., 2023), and *Variovorax* (Cabrera-Orozco et al., 2017; Billet et al., 2019). These results reinforce the idea that these key genera may be central to the biodegradation process in nutrient-limited conditions. Interestingly, the microbial community in the presence of ammonium exhibited less diversity, yet this was associated with more efficient atrazine degradation. Our findings suggest that the presence of ammonium facilitated microbial growth, which could enable better adaptation for subsequent atrazine utilization. Some genera, including *Klebsiella* (Yang et al., 2010; Cabrera-Orozco et al., 2017) and *Stenotrophomonas* (Liu et al., 2023), were previously found to be responsible for atrazine degradation. These results highlight the role of nitrogen sources in shaping the functional potential of microbial communities and their capacity for pollutant degradation. Moreover, the nitrogen sources significantly affected the viral community composition and viral lifestyles. The relative abundance of *Peduoviridae* and temperate virus in the absence of the exogenous nitrogen source was much higher than that in the presence of ammonium (Figures 4a,b).

Furthermore, viral amendments had considerable influence on both the bacterial and viral community composition and diversity. The addition of either lytic or temperate viruses altered microbial community dynamics, with lytic viruses potentially enhancing microbial turnover through host cell lysis and nutrient release (Kuzyakov and Mason-Jones, 2018). In particular, the relative abundance of *Peduoviridae*, particularly those infecting *Firmicutes*, was higher under nitrogen-limiting conditions (Figure 4a), suggesting a link between viral presence and enhanced microbial activity. These shifts, along with the observed changes in microbial community diversity, support the notion that viruses can act as key modulators of microbial composition structure in contaminated environments.

### 4.4 The critical roles of nitrogen sources and viruses in mediating the metabolic pathways

Most previously characterized atrazine-degrading bacteria harbor the genes of *atzA/trzN*, *atzB*, *atzC*, *atzD*, *atzE*, and *atzF* for complete mineralization of atrazine (Billet et al., 2019). Here, *atzA/trzN* were not detected in metagenomes, but *atzB*, *atzC*, *atzD*, *atzE*, and *atzF* were found. It may be due to the limitation in sequencing depth or falling into unassembled/short contigs (Supplementary Table S1). In addition, the expression of genes related to degradation is often activated by the presence of xenobiotics. Thus, the failure to detect *atzA/trzN* may be due to the lack of atrazine that was removed in the culture medium. These genes were carried by different bacteria, suggesting the synergistic degradation pathway that was commonly the degradation pathway for atrazine. The presence of nitrogen sources notably modulated the expression of atrazine degradation genes in the microbial community. Atrazine degradation genes can be repressed in the presence of a simple nitrogen source like ammonium chloride (Sharma et al., 2019). Here, fewer atrazine degradation genes were detected in the presence of ammonium (Figure 7b). The inhibition of atrazine degradation by ammonium observed in this study further supports the idea that nitrogen status can regulate the expression of catabolic pathways for xenobiotics.

Viruses, particularly temperate viruses, were found to harbor AMGs, which could significantly influence microbial metabolic pathways (Sun et al., 2023). In this study, AMGs associated with the carbon, sulfur, and phosphorus metabolism suggested the virus played a role in the nutrient cycling in the enrichment cultures. Temperate viruses possessed a greater abundance and diversity of AMGs while playing a pivotal role in mediating the microbial community composition and function. Thus, these virus-encoded AMGs likely contributed to host fitness and metabolism in contaminated environments and should be considered during the bioremediation of contaminated soils (Zheng et al., 2022). For example, temperate viruses under higher chromium-induced stress carried more AMGs regulating microbial heavy metal detoxification and resistance (Huang et al., 2021). Moreover, some AMGs were found to be related to carbon and phosphorus metabolic pathways, which may have enhanced the metabolic activity of the enriched communities. The relative abundance of most key functional genes was greater in the treatment with ammonium and intracellular virus, indicating that the nutrient metabolism was more active. Here, the relative abundance of nitrogen metabolism genes was significantly higher with the addition of temperate-dominated viruses. It was conjectured that temperate viruses may enhance the depletion of ammonium in the medium and relieve the nitrogen inhibition for atrazine degradation. The higher abundance of atrazine degradation genes with the addition of temperate-dominated viruses also suggested the viral-mediated microbial turnover may enhance biodegradation potential. Some studies have shown that AMGs potentially associated with the degradation of organic pollutants have also been identified (Sun et al., 2023; Chao et al., 2023; Xia et al., 2023; Yuan et al., 2023). However, atrazine degradation genes were not found in AMGs here. Due to the limited viral contigs with high quality, the viral community and AMGs may be underestimated, and pollutant-related genes were not analyzed and annotated as the virus-encoded AMGs. These findings highlight the potential for viruses to not only influence microbial diversity but also actively mediate microbial metabolism in ways that support bioremediation processes.

## 5 Conclusion

This study elucidates the complex interactions between nitrogen sources, viral communities, and microbial dynamics in the biotransformation of atrazine, contributing new insights into the role of microbial-viral interactions in pollutant biodegradation. Both lytic and temperate viruses interact with microbial communities to enhance atrazine degradation by different strategies. Lytic viruses, through selective host lysis, promote nutrient cycling by releasing cellular components, thereby stimulating microbial activity under nitrogen-limiting conditions. In contrast, temperate viruses, via the expression of AMGs, directly influence microbial metabolism, particularly nitrogen and carbon cycles, further enhancing atrazine degradation. Importantly, this study underscores the dynamic relationship between microbial and viral communities and their potential for application in bioremediation strategies. The identification of key bacterial taxa involved in atrazine degradation, particularly in virus-amended treatments, points to the importance of synergistic interactions in microbial consortia, further advancing our understanding of pollutant degradation in complex environmental systems. These findings contribute to a growing body of knowledge on how nutrient availability and viral infections can be harnessed to optimize bioremediation processes in agricultural and contaminated soils. Future studies, incorporating more comprehensive viral metagenomic analyses, are needed to fully explore the extent to which viruses carry genes involved in the degradation of organic pollutants, including atrazine.

## Data Availability

The original contributions presented in the study are publicly available. This data can be found at: https://www.ncbi.nlm.nih.gov, accession number PRJNA1176382 and PRJNA1177983.
