# Supplementary material for: The interplay of nitrogen sources and viral communities in the biodegradation of atrazine in agricultural soils
Source: Front Microbiol. 2025 Jul 16;16:1645559. doi: 10.3389/fmicb.2025.1645559 (PMC12307285; doi:10.3389/fmicb.2025.1645559)
Supplement: Supplementary file 1 [file Data_Sheet_1.pdf]

The Interplay of Nitrogen Sources and Viral Communities in the Biodegradation of Atrazine in Agricultural Soils

Yongfeng Wang[1,2], Mark Radosevich[3], Lu Yang[4], Ying Zhang[1], Ninghui Xie[1,2], Xiaolong Liang[1,2]*

1 Key Laboratory of Pollution Ecology and Environmental Engineering, Institute of Applied Ecology, Chinese Academy of Sciences, Shenyang 110016, China

2 CAS Key Laboratory of Forest Ecology and Silviculture, Institute of Applied Ecology, Chinese Academy of Sciences, Shenyang 110016, China

3 Department of Biosystems Engineering and Soil Science, The University of Tennessee, Knoxville, TN 37996, U.S.A.

4 The Benioff Center for Microbiome Medicine, University of California San Francisco, San Francisco, CA 94080, U.S.A.

* Corresponding author: Xiaolong Liang (liangxlucas@outlook.com)

Supplementary materials

Table S1. Summary of sequencing quality and assembly

Figure S1. Degradation of atrazine (solid line) and microbial growth (dash line) in sterilized treatment. All data show the mean of three replicates with standard deviation.

Figure S2. Transformation product of atrazine.

Figure S3. Relative abundance of top 10 microbial phyla based on metagenomic sequencing.

Figure S4. Relative abundance of atrazine degradation genes. CV_C, CV_{EX}, and CV_{IN} denote no virus, extracellular and intracellular virus without exogenous nitrogen other than atrazine; AV_C, AV_{EX}, and AV_{IN} denote no virus, extracellular and intracellular virus with ammonium amendments. Data show the mean of three replicates with standard deviation, and different lowercase letters among treatments denote significant differences (ANOVA, $p < 0.05$).

Figure S5. Relative abundance of top 10 KEGG pathways (L3) of virus-encoded AMGs based on metagenomic sequencing.

Table S1 Summary of sequencing quality and assembly

Sample*	Raw Reads $\times 10^7$	Raw Bases $\times 10^{10}$	Clean Reads $\times 10^7$	Clean Bases $\times 10^{10}$	Scaffold number $\times 10^5$	Scaffold total length (bp) $\times 10^8$	Scaffold average length (bp) $\times 10^3$	Scaffold N50 length (bp) $\times 10^3$
CV_C1	7.71	1.16	6.81	1.01	0.96	2.07	2.16	5.29
CV_C2	7.35	1.10	6.56	0.98	0.97	2.06	2.12	4.94
CV_C3	9.68	1.45	6.43	0.96	1.00	1.99	2.00	4.55
$CV_{EX}1$	8.43	1.27	7.42	1.10	1.19	2.44	2.05	5.32
$CV_{EX}2$	8.49	1.27	7.52	1.12	1.15	2.41	2.10	6.12
$CV_{EX}3$	7.05	1.06	6.22	0.93	1.07	2.28	2.13	5.58
$CV_{IN}1$	6.65	1.00	5.85	0.87	0.91	2.03	2.23	5.65
$CV_{IN}2$	7.42	1.11	6.52	0.97	0.97	2.20	2.27	6.35
$CV_{IN}3$	7.52	1.13	6.63	0.99	1.04	2.30	2.21	6.01
AV_C1	7.83	1.18	6.88	1.03	2.46	3.86	1.57	2.19
AV_C2	6.71	1.01	5.95	0.89	2.29	3.54	1.54	2.07
AV_C3	7.09	1.06	6.21	0.93	2.41	3.55	1.47	1.86
$AV_{EX}1$	7.47	1.12	6.59	0.98	2.27	3.67	1.62	2.24
$AV_{EX}2$	7.25	1.09	6.46	0.96	2.26	3.73	1.65	2.37
$AV_{EX}3$	7.05	1.06	6.31	0.94	2.21	3.58	1.62	2.26
$AV_{IN}1$	7.60	1.14	6.78	1.01	1.76	2.69	1.53	2.20
$AV_{IN}2$	8.21	1.23	7.27	1.09	2.07	3.03	1.46	1.89
$AV_{IN}3$	7.65	1.15	6.66	0.99	1.69	2.69	1.59	2.47

*CV_C, CV_{EX}, and CV_{IN} denote no virus, extracellular and intracellular virus without exogenous nitrogen other than atrazine; AV_C, AV_{EX}, and AV_{IN} denote no virus, extracellular and intracellular virus with ammonium amendments.

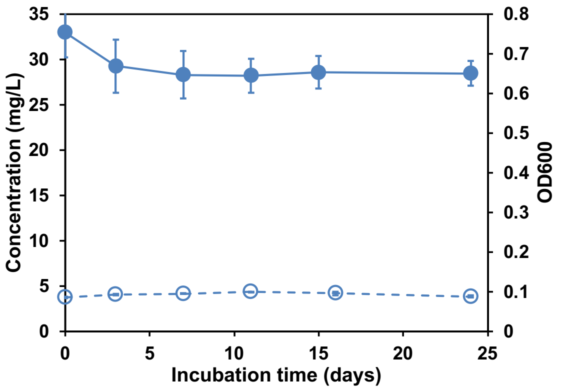

Figure S1. Degradation of atrazine (solid line) and microbial growth (dash line) in sterilized treatment. All data show the mean of three replicates with standard deviation.

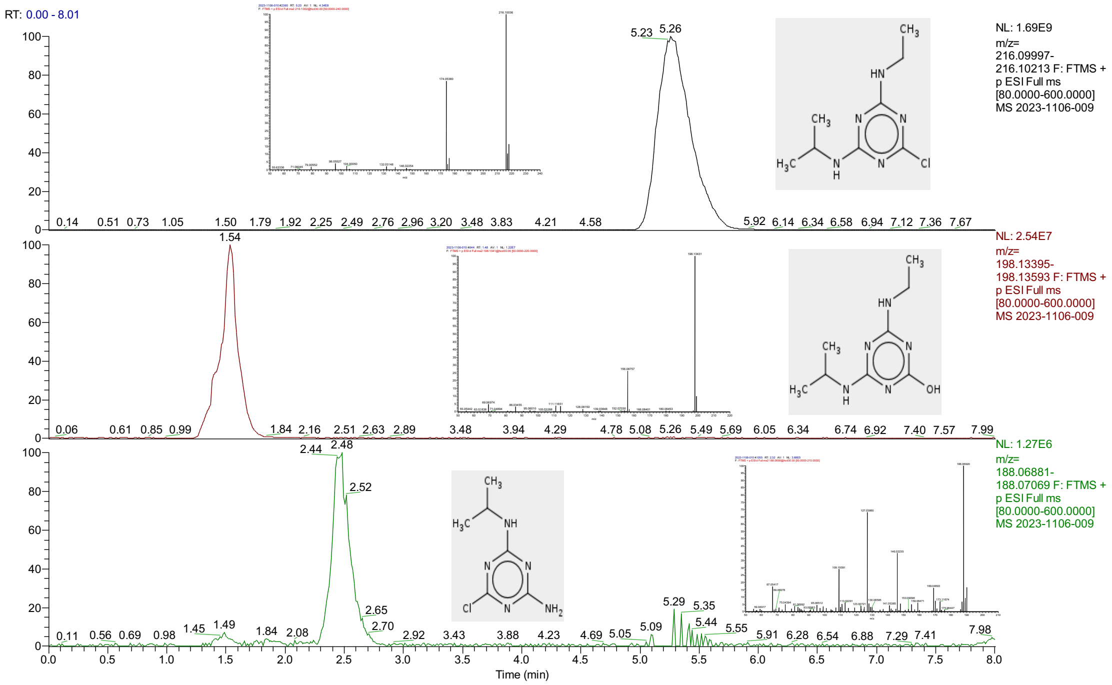

Figure S2. Transformation product of atrazine.

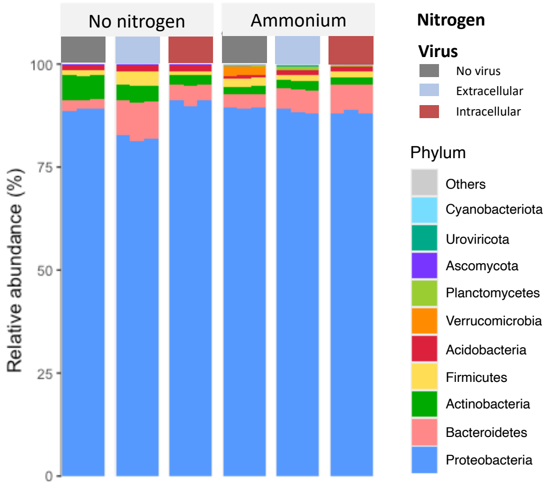

Figure S3. Relative abundance of top 10 microbial phyla based on metagenomic sequencing.

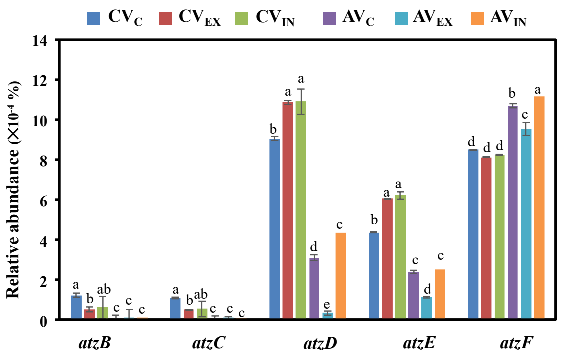

Figure S4. Relative abundance of atrazine degradation genes. CV_C, CV_{EX}, and CV_{IN} denote no virus, extracellular and intracellular virus without exogenous nitrogen other than atrazine; AV_C, AV_{EX}, and AV_{IN} denote no virus, extracellular and intracellular virus with ammonium amendments. Data show the mean of three replicates with standard deviation, and different lowercase letters among treatments denote significant differences (ANOVA, $p < 0.05$).

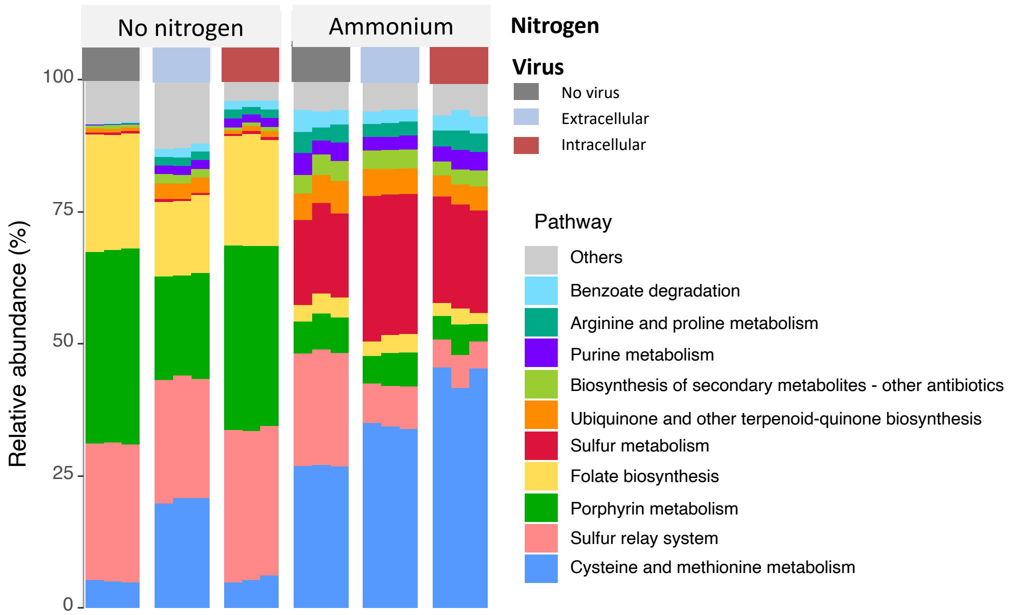

Figure S5. Relative abundance of top 10 KEGG pathways (L3) of virus-encoded AMGs based on metagenomic sequencing.
